# Household Consumption of Adequately Iodized Salt: A Multi-Country Analysis of Socioeconomic Disparities

**DOI:** 10.3390/nu16213787

**Published:** 2024-11-04

**Authors:** Daniela M. Sáez-Ramírez, Horacio Chacon-Torrico, Akram Hernández-Vásquez

**Affiliations:** 1 Facultad de Ciencias de la Salud, Universidad Científica del Sur, Lima 15067, Peru; 100097434@cientifica.edu.pe; 2 Facultad de Ciencias de la Salud, Universidad Continental, Huancayo 12001, Peru; hchacon@continental.edu.pe; 3 Centro de Excelencia en Investigaciones Económicas y Sociales en Salud, Vicerrectorado de Investigación, Universidad San Ignacio de Loyola, Lima 15024, Peru

**Keywords:** iodine, diet, health surveys, sodium chloride, socioeconomic factors

## Abstract

Background: Despite global efforts to promote universal salt iodization, iodine deficiency remains a public health issue in developing countries. Objectives: This study assessed the proportion and sociodemographic characteristics of households consuming adequately iodized salt in 49 low- and middle-income countries. Methods: Data from DHS surveys of 49 low- and middle-income countries (2005–2021) were used to analyze household iodized salt prevalence. R version 4.0 was employed for statistical analyses. A random-effects meta-analysis was conducted to estimate overall and regional prevalence. Results: We found that 83.4% of households consume adequately iodized salt, although with high heterogeneity (*I*^2^ = 100.0%). The East Asia and Pacific and the Europe and Central Asia regions showed high consumption rates of 87.6% and 87.7%, respectively, while Latin America and the Caribbean presented a significantly lower proportion of 30.8%. Conclusions: The study highlights the need for enhanced public health strategies to increase iodized salt consumption, especially in low-income and rural households. Addressing disparities in access, education, and affordability is crucial for improving iodine intake and preventing deficiency disorders, particularly among vulnerable populations like children and pregnant women.

## 1. Introduction

Iodine deficiency remains a persistent challenge for global public health. Despite ongoing efforts, in 2019, the global prevalence of this deficiency remained at 2.4%, according to data from the Global Burden of Disease [1]. For example, in Brazil, the prevalence of iodine deficiency among pregnant women is 40% [2] and in Tibet, the prevalence in the general population is 25.6% [3]. The severe effects of this deficiency, including iodine deficiency disorders, goiter, cretinism, and heart failure, pose a considerable risk to public health [4,5,6]. In particular, during pregnancy, iodine deficiency can lead to hypothyroidism, with devastating consequences for the fetus, such as delayed cognitive development or miscarriage [4,6,7]. Additionally, in the early years of life, iodine deficiency can cause intellectual disability, impacting child development [8,9], and during childhood and adolescence, it can affect both physical and cognitive development [8].

Given the far-reaching and severe health consequences of iodine deficiency, addressing this issue on a global scale is crucial. As part of the global response, international organizations such as the World Health Organization (WHO) and the United Nations Children’s Fund (UNICEF) have promoted universal salt iodization policies since 1994, establishing a standard of 20 to 40 parts per million (ppm) of iodine in salt used for food preparation. This strategy has shown promising results in countries such as China, Peru, and India [10,11,12]. This demonstrates that with a strong commitment, proper long-term monitoring, and the implementation of realistic strategies tailored to the specific circumstances of each country, it is possible to ensure that the majority of the population has access to iodized salt. Indeed, by 2020, 89% of households worldwide were consuming iodized salt, and the number of countries involved increased from 67 in 1993 to 118 in 2020 [13,14]. However, despite the success in several countries, the global implementation of salt iodization programs remains inconsistent, with significant challenges in regions such as Eastern and Southern Africa, where access to adequately iodized salt remains limited [13]. Despite the progress made, there are still countries and population groups that do not consume adequately iodized salt and, therefore, require ongoing attention.

The primary objective of our study is to provide a global and comparative perspective of the consumption of adequately iodized salt, addressing the barriers and trends observed across different sociodemographic contexts. While previous research has focused on individual countries such as Tunisia [15], Ethiopia [16], Bangladesh [17], and Peru [18], and there have been regional studies in Sub-Saharan Africa that have examined iodized salt consumption in 31 and 11 countries, respectively [19,20], these studies offer limited perspectives. For example, UNICEF has also leveraged data from Demographic and Health Surveys (DHS) and Multiple Indicator Cluster Surveys (MICS) to gain insight into the state of salt iodization in certain regions [13]. However, to date, no comprehensive analyses have been conducted at a macro level to identify broader patterns or common barriers across multiple countries. While these national studies provide valuable insights, they are often limited in their scope, focusing on specific countries or regions. This presents a gap in the literature, as it becomes difficult to compare global patterns and address disparities comprehensively. To bridge this gap, our study integrates data from 49 countries, offering a more holistic and comparative assessment of iodized salt consumption across varying socioeconomic contexts. This comparative approach not only allows for the identification of gaps in policy implementation, but also evaluates the effectiveness of interventions in specific contexts, contributing significant value to global public health strategies.

Current evidence suggests that there are significant disparities in iodized salt consumption, influenced by sociodemographic factors, such as wealth, education, and geographic location. An analysis conducted by UNICEF in 2020 [13], using data from MICS and DHS of 82 countries, revealed that 60% of households in the highest wealth quintile consumed adequately iodized salt. In Ethiopia, some studies show that the wealthiest quintile is twice as likely to consume iodized salt compared to the lower quintiles [21]. Similarly, studies conducted in China indicate that individuals with lower incomes have a significantly higher risk of not having access to iodized salt [16,22]. Additionally, it has been observed that, in general, urban households have a higher consumption of iodized salt compared to rural households [22,23], and in Sub-Saharan Africa, households with educational levels ranging from primary to higher education show a greater tendency to use iodized salt than those without education [19,22]. Finally, a recent systematic review found that women with higher education levels are almost twice as likely to consume iodized salt compared to their less-educated counterparts [24].

Despite monitoring efforts and the implementation of policies, the systematization of information on iodized salt consumption remains a challenge. In 2010 the WHO introduced the “Conceptual Framework for Action on the Social Determinants of Health”, which addresses the conditions in which individuals are born, grow, live, work, and age. These conditions are profoundly shaped by the distribution of power, wealth, and resources within society [25]. Within this framework, health equity is defined as the absence of avoidable and remediable differences in health among different social groups, emphasizing the importance of social justice and human rights as fundamental pillars for reducing these disparities. Furthermore, power dynamics are considered crucial in addressing health inequities, necessitating political interventions that engage both disadvantaged communities and governments in fostering structural changes that enhance equity in healthcare delivery.

Regarding universal salt iodization, its implementation may present significant challenges across different socioeconomic contexts. Factors such as cultural differences and economic inequalities, particularly in vulnerable communities located in remote areas, can limit its effectiveness [26]. Therefore, public policies aimed at salt iodization must take into account the social determinants of health and adapt strategies to ensure equitable access for marginalized populations. The design of these policies should focus not only on the technical aspects of implementation, but also on addressing the structural barriers that perpetuate inequalities in access to essential health interventions [27].

In light of this situation, this study assessed the proportion and sociodemographic characteristics of households consuming adequately iodized salt in 49 low- and middle-income countries. This global analysis sought to identify gaps in policy implementation and propose interventions that are effective within specific socioeconomic contexts.

## 2. Materials and Methods

Data sets from the DHS [28], collected between 2005 and 2021, were used as secondary data sources. These surveys, which are available through the DHS Program API, covered 49 low- and middle-income countries (LMIC). The countries were classified according to the regional categories of the World Bank: East Asia and the Pacific, Europe and Central Asia, Latin America and the Caribbean, Middle East and North Africa, South Asia, and Sub-Saharan Africa [29].

To obtain microdata, statistical analyses were conducted in R version 4.0 (2023). The ggplot2 package was used to create the figures, and the rdhs package in R was utilized to automate the retrieval of DHS data [30]. Readers interested in the methodology of the DHS surveys can review detailed information, including complex sampling procedures, on the DHS Program website [28]. It is important to acknowledge the limitations of these surveys, such as reporting and recall biases, particularly for retrospective data, as described in the original DHS methodological documentation.

Table 1 presents the countries and their respective regions, grouped according to their World Bank categories [29], as well as the years in which the DHS surveys were conducted [28].

### 2.1. Eligibility Criteria

The study was based exclusively on the most recent DHS data sets from 49 countries that included information on the prevalence of iodized salt in households. The analysis was conducted at the household level, and the inclusion criteria required that the surveys contained the following variables of interest: household iodine testing, wealth index quintiles, educational level, and area of residence. Surveys that lacked data on salt iodine testing or did not have complete information for the three variables described were excluded.

### 2.2. Variables

The dependent variable focused on the household iodine test results, captured by two DHS variables: HV234 and HV234a. The HV234 variable was adapted such that iodine levels below 15 ppm were coded as ‘0’, while levels of 15 ppm or higher were coded as ‘1’. In cases in which HV234 was unavailable, we used HV234a, which qualitatively reported the presence or absence of iodine. This variable was recoded as ‘1’ if iodine was present and ‘0’ if it was absent. Households without iodine test data were excluded from the analysis. To account for sociodemographic variations in each country, stratification variables, such as type of residential area (urban or rural), wealth index quintiles (from poorest to richest), and educational level (no education, primary, secondary, and higher education), were employed.

### 2.3. Data Analysis

Data extraction from the DHS was conducted using R version 4.0, with the rdhs package facilitating access to the DHS data sets [31]. We obtained overall and country-specific estimates of the prevalence of iodized salt use using the srvyr package in R, accounting for the complex survey design and weights. This package allowed us to account for the stratified multistage sampling design, ensuring that our estimates were nationally representative. Weighted dumbbell plots were created to visualize within- and across-country variations in the prevalence of adequately iodized household salt consumption, stratified by area of residence (urban or rural), wealth quintile, and the highest educational attainment of the household head. Countries were sorted from top to bottom based on prevalence, with those having the lowest prevalence of iodized salt consumption at the top and those with the highest prevalence at the bottom. The x-axis was transformed using a square root scale to emphasize larger variations in prevalence at higher values. All estimates included 95% confidence intervals (CI).

Next, weighted estimates for iodized salt testing were obtained from the individual DHS data sets for each country. A random-effects meta-analysis was conducted to derive both the overall and region-specific prevalence of iodized salt use. This approach was chosen to account for the inherent heterogeneity between countries due to differing socioeconomic and regional factors, which a fixed-effects model would not accommodate. Forest plots were generated using the meta package to display the proportions for each country, their corresponding weights, and the aggregated prevalence within each region, along with their 95% CIs. To assess the stability and robustness of the meta-analysis results, a sensitivity analysis was performed by excluding one country at a time from the meta-analysis.

To promote transparency and reproducibility, the entire analytical code is publicly accessible in a Zenodo repository [32].

### 2.4. Ethical Considerations

The Institutional Research Ethics Committee of the Universidad Científica del Sur approved this study (registration code: POS-61-2022-00099), which will serve as a partial requirement for one of the authors to obtain a master’s degree.

## 3. Results

**Figure 1:**
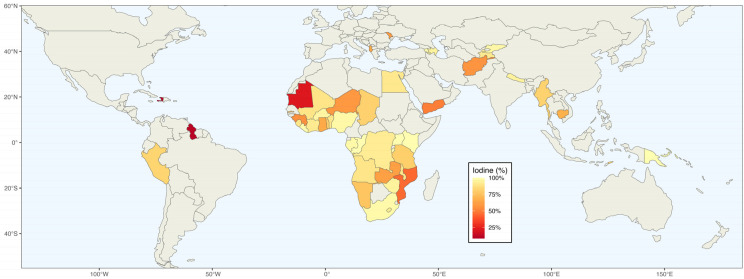
The DHS surveys included in the study.

DHS surveys from 49 countries were included in the meta-analysis, as shown in Figure 1. These countries span various regions, with four from East Asia and the Pacific, six from Europe and Central Asia, three from Latin America and the Caribbean, two from the Middle East and North Africa, two from South Asia, and thirty-two from Sub-Saharan Africa, with data ranging from 2005 (Moldova) to 2021 (Peru). The final data set includes information from 583,145 interviewed households, of which 468,463 consume adequately iodized salt. The global proportion of households consuming adequately iodized salt is 83.4% (95% CI: 77.0–88.9%) according to a random-effects model, highlighting significant heterogeneity (*I*^2^ = 100.0%). This variability is reflected in the substantial differences between regions of the world.

**Figure 2:**
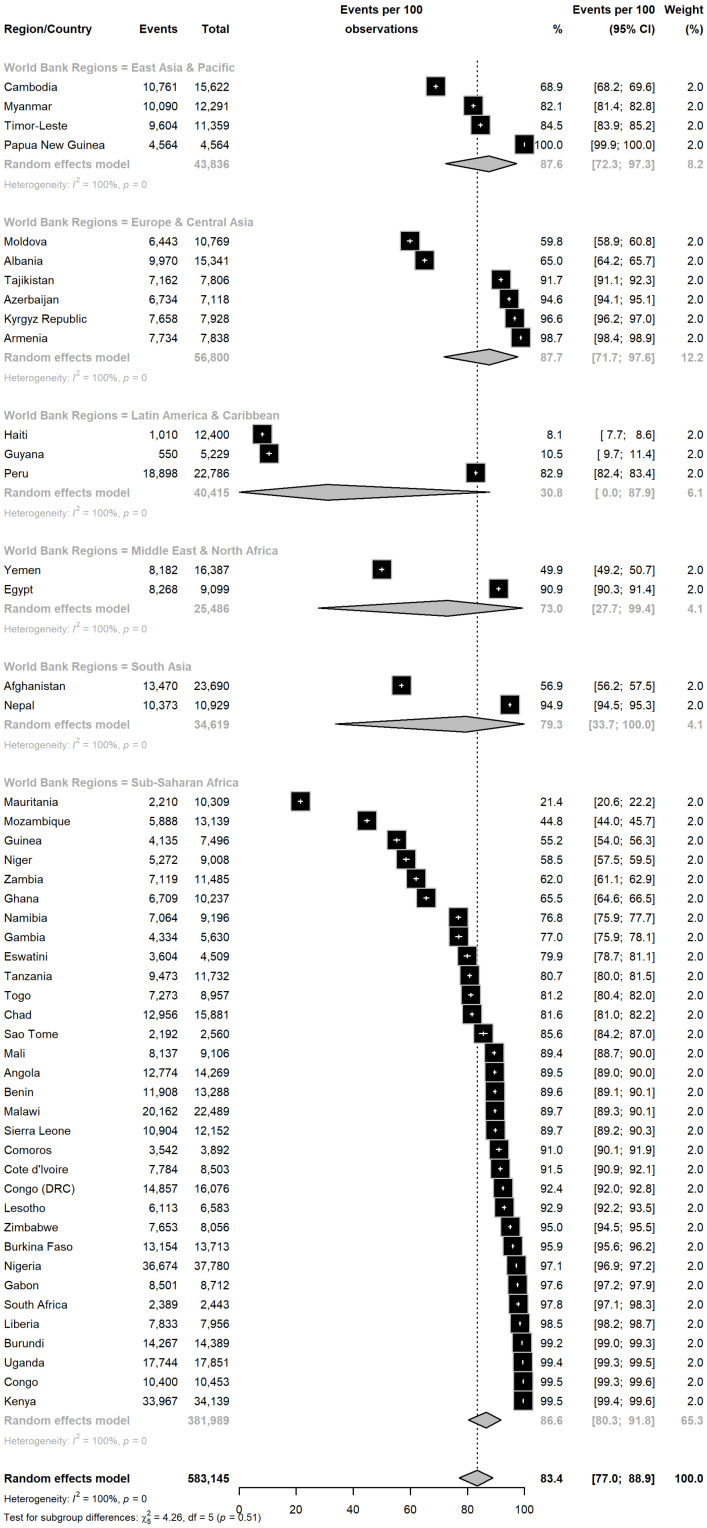
The prevalence of adequately iodized salt consumption in different countries and regions, as classified by the World Bank.

In Europe and Central Asia, the proportion of households consuming adequately iodized salt is 87.7% (95% CI: 71.7–97.6%). In East Asia and the Pacific, the proportion is 87.6% (95% CI: 72.3–97.3%). Sub-Saharan Africa shows a proportion of 86.6% (95% CI: 80.3–91.8%). South Asia reports 79.3% (95% CI: 33.7–100.0%), while the Middle East and North Africa show 73.0% (95% CI: 27.7–99.4%). In contrast, Latin America and the Caribbean present a considerably lower proportion of 30.8% (95% CI: 0.0–87.9%) (Figure 2). The sensitivity analysis showed that the estimate varied between 83% and 85%; the results are presented in the Supplementary Material.

Figure 2 details the country-specific estimates regarding the proportion of households consuming adequately iodized salt. Among the countries with a proportion of 90% or higher, 21 nations stand out. In East Asia and the Pacific, Papua New Guinea reports 100%. In Sub-Saharan Africa, the following countries are highlighted: Kenya (99.5%), Congo (99.5%), Uganda (99.4%), Burundi (99.2%), Liberia (98.5%), South Africa (97.8%), Gabon (97.6%), Nigeria (97.1%), Burkina Faso (95.9%), Zimbabwe (95%), Lesotho (92.9%), Democratic Republic of the Congo (92.4%), Ivory Coast (91.6%), and Comoros (91%). In Europe and Central Asia, Armenia (98.7%), Kyrgyzstan (96.6%), Azerbaijan (94.6%), and Tajikistan (91.7%) also show high proportions. In South Asia, Nepal stands out with 94.9%. In the Middle East and North Africa, Egypt reports 90.9%. Among the countries with the lowest proportions, Sub-Saharan Africa includes Mauritania (21.4%), while in Latin America and the Caribbean, Guyana reports 10.5% and Haiti 8.1%.

**Figure 3:**
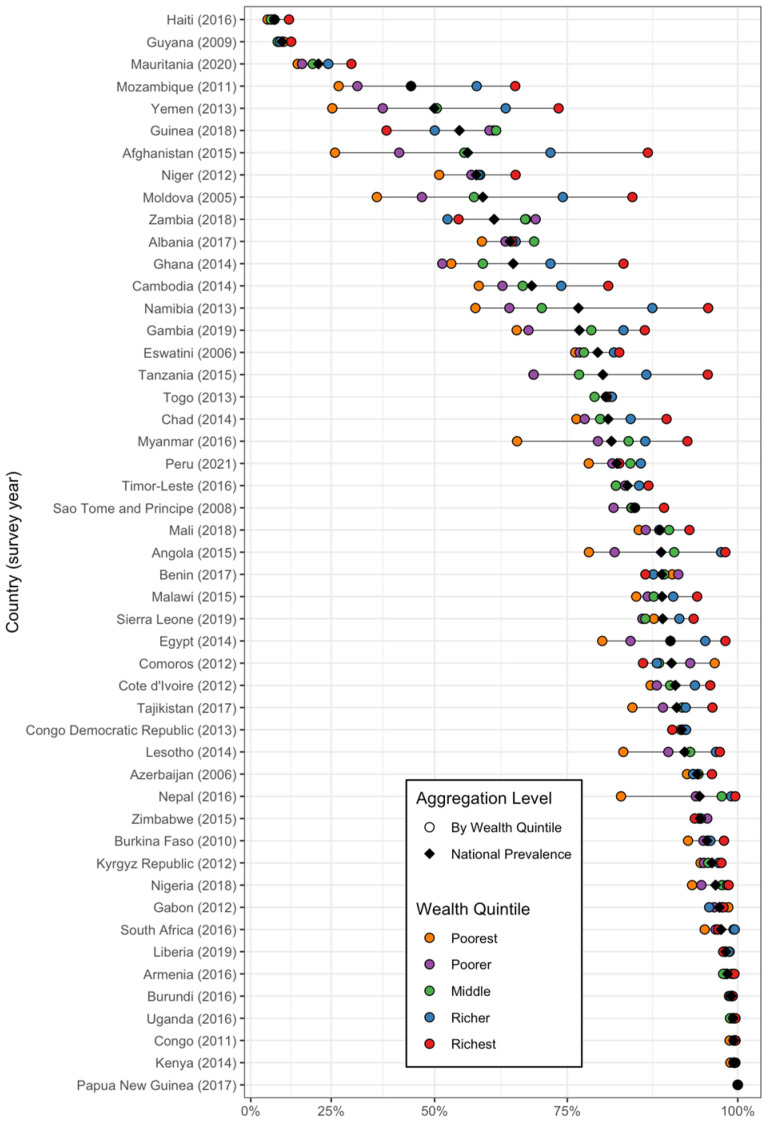
The prevalence of adequately iodized salt consumption in households by country, national prevalence, and wealth quintiles. The circles represent iodized salt consumption levels segmented by wealth quintiles, with color coding as follows: orange for the poorest quintile, purple for poorer, green for middle, blue for richer, and red for the richest. The black diamond indicates the national prevalence.

In relation to the wealth quintile (Figure 3), the higher wealth quintiles generally show the highest proportion of households consuming adequately iodized salt. In contrast, the lower quintiles display, on average, the lowest proportion, with exceptions in Sub-Saharan African countries such as Kenya, Congo, Uganda, Burundi, Gabon, Zimbabwe, Democratic Republic of the Congo, Nigeria, Benin, Togo, and Eswatini, as well as Papua New Guinea, located in East Asia and the Pacific. In Europe and Central Asia, particularly in Armenia, Kyrgyzstan, and Azerbaijan, as well as in Haiti and Guyana in Latin America and the Caribbean, no marked differences can be observed between wealth quintiles.

**Figure 4:**
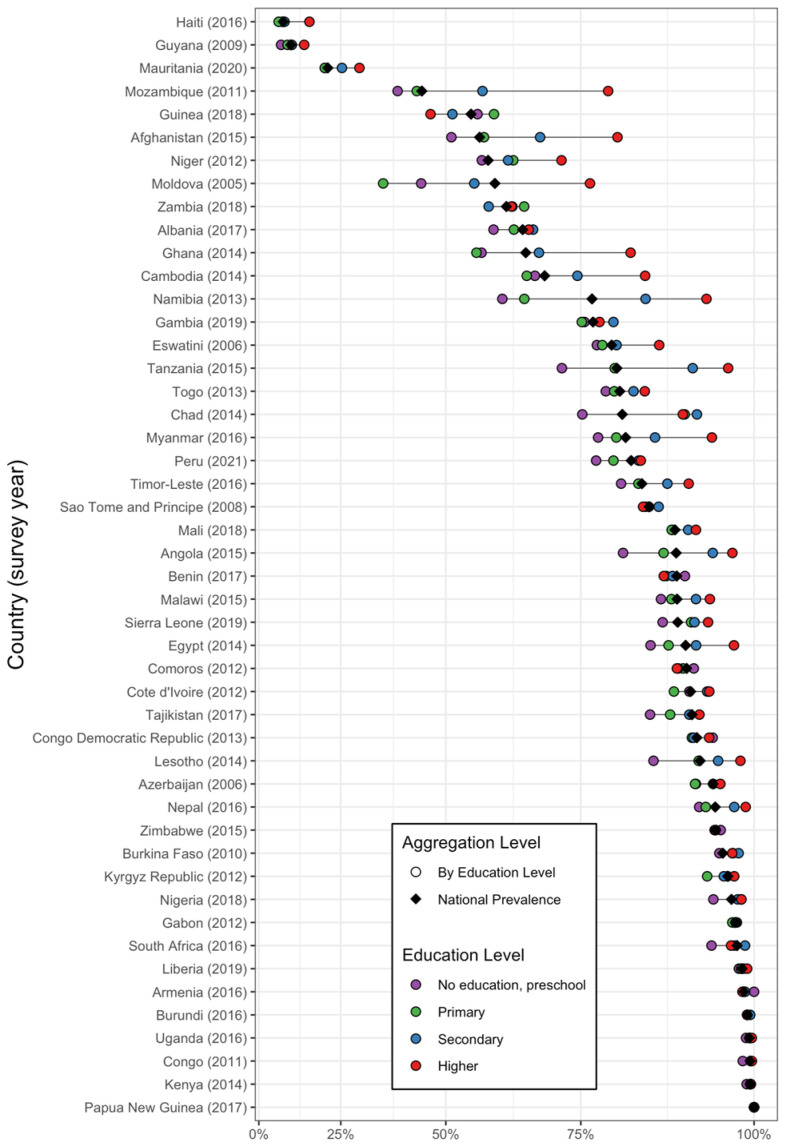
The prevalence of adequately iodized salt consumption in households by country, national prevalence, and educational level. The circles represent iodized salt consumption levels segmented by education levels, with color coding as follows: purple for those with no formal education or only preschool, green for primary education, blue for secondary education, and red for higher education. The black diamond indicates the national prevalence.

Figure 4 illustrates that households with higher education levels tend to have a greater proportion of iodized salt consumption in most countries. However, similar values can be observed in countries such as Papua New Guinea, located in East Asia and the Pacific; Armenia in Europe and Central Asia; and the following countries in Sub-Saharan Africa: Kenya, Congo, Uganda, and Burundi. The gap is more pronounced in Myanmar and Cambodia, located in East Asia and the Pacific; Tanzania and Afghanistan, in South Asia; Mozambique, Ghana, and Namibia, located in Sub-Saharan Africa; and Moldova in Europe and Central Asia.

**Figure 5:**
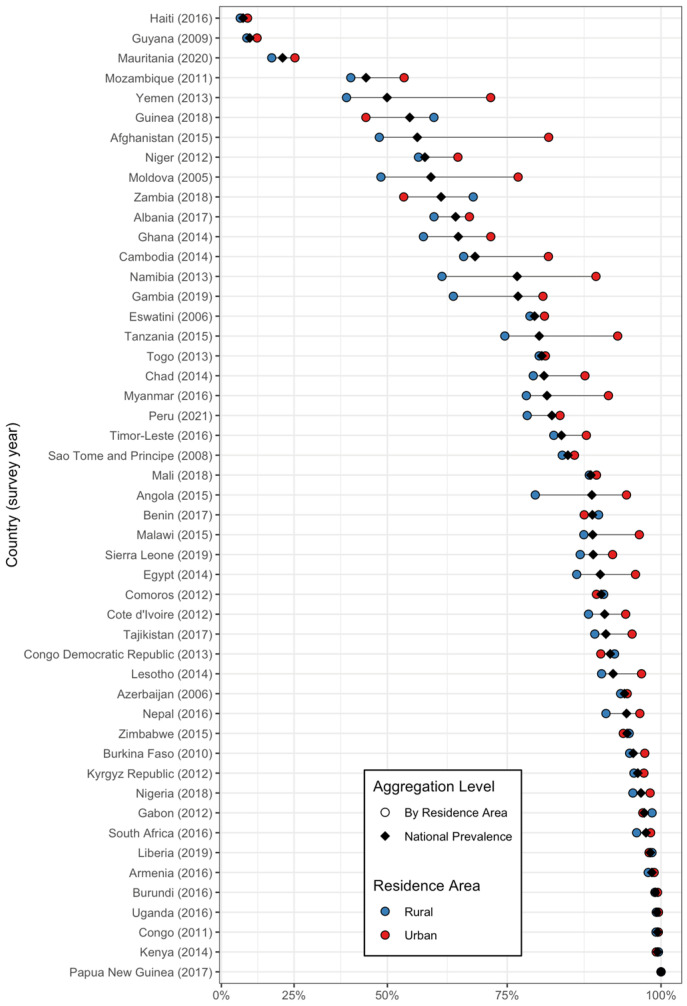
The prevalence of adequately iodized salt consumption in households by country, national prevalence, and area of residence. The circles represent iodized salt consumption levels segmented by residence area, with color coding as follows: blue for rural areas and red for urban areas. The black diamond denotes the national prevalence.

Regarding the place of residence (Figure 5), in most countries, notable differences can be observed between rural and urban areas in terms of the prevalence of adequately iodized salt consumption. Conversely, in Mozambique, Ghana, Namibia, Gambia, Tanzania, and Angola, located in Sub-Saharan Africa; Afghanistan in South Asia; Moldova in Europe and Central Asia; Egypt and Yemen in the Middle East and North Africa; and Myanmar in East Asia and the Pacific, a higher proportion of consumption can be observed in rural households compared to urban households. In countries such as Papua New Guinea in East Asia and the Pacific; Kenya, Congo, Uganda, Burundi, and Liberia, located in Sub-Saharan Africa; and Armenia in Europe and Central Asia, the national prevalence is approximately midway between the urban and rural prevalence, suggesting a relatively balanced distribution.

## 4. Discussion

This study aimed to determine the proportion and sociodemographic characteristics of households consuming adequately iodized salt. The results show that it is consumed in 83.4% of the households surveyed, meaning that on average, 8 out of 10 households use adequately iodized salt. Although the WHO considers universal salt iodization to be a cost-effective intervention [33], with an expenditure of USD 0.02–0.05 per person per year, its implementation remains insufficient in several countries. As a result, a significant percentage of the world population consumes salt with inadequate iodine levels, including the most vulnerable groups, such as pregnant women and children.

The UNICEF ‘State of the World’s Children 2019’ report noted that 86% of households worldwide incorporated iodized salt into their diets [9]. Subsequently, in the 2021 edition of the same report, this figure increased to 89% [13,34]. Our research, which covers surveys from 2005 to 2021, indicates that 83.4% of households have adopted the practice of consuming adequately iodized salt. It is important to highlight that the definition of the indicator ‘adequately iodized salt’ changed in 2016. Previously, the indicator referred to households consuming salt with an iodine concentration between 20 and 40 parts per million (ppm) [33]. After that date, UNICEF indicators refer to households consuming salt with a minimum iodine content above 0 ppm. Despite the variability in data collected over different years and changes in the indicator’s definition over time, a clear increase in iodized salt consumption is evident. However, the gap that must be closed to reach the goal of 90% of households consuming iodized salt still persists.

There have been several studies conducted in countries such as Tunisia [15], Bangladesh [17], Peru [18], and Ethiopia [16,35], as well as in regions like Sub-Saharan Africa [19,20], that share similarities with our approach in using Demographic and Health Surveys (DHS) to evaluate iodized salt consumption. Like our study, these works also take into account key sociodemographic factors, including wealth index, education level, and residential area. A noteworthy aspect of the research conducted in Sub-Saharan Africa and Ethiopia is the inclusion of exposure to mass media, such as television and radio [19,20], which is particularly relevant for assessing the impact of large-scale educational campaigns aimed at improving iodized salt consumption. This approach leverages the influence of mass media as a powerful tool for raising awareness and educating populations, especially in rural communities with limited access to healthcare services. For example, a study in Ethiopia demonstrated that media exposure was directly linked to a higher likelihood of iodized salt consumption [35]. Such strategies can be crucial in achieving greater success in the implementation of universal salt iodization policies in low-income countries.

Regarding the wealth quintile, households in the higher quintiles show a significantly greater proportion of iodized salt consumption. Various studies in Sub-Saharan Africa [19,20,23], Bangladesh [17], and Ethiopia [16,21,22] corroborate these findings. Although salt iodization is an affordable strategy for preventing iodine deficiency disorders, it remains an issue in low-income households. For example, in Ethiopia, households with monthly incomes above USD 60 are four times more likely to consume iodized salt compared to lower-income households [22]. A similar pattern is observed in India, where families with monthly incomes of USD 10 or more are more likely to have access to iodized salt [36]. This disparity may be due to the lower likelihood of individuals in lower quintiles purchasing iodized salt [19]. This difference in access is partly attributable to cost differences, as the annual cost of salt iodization is approximately USD 0.05 per person per year [37], leading low-income families to opt for alternatives, such as local or retail channel salts [9], which lack government-regulated control and have shown inconsistent production in terms of added iodine content. In Bangladesh, between 2001 and 2002, it was observed that out of 122 factories analyzed in four districts, none maintained adequate iodine levels in their products, and most mills incorporated amounts below the required standards [38]. These findings support the notion that while salt iodization can be considered an economically viable option, its applicability and effectiveness vary significantly according to the socioeconomic realities of each country. Ultimately, the results underscore a direct relationship between the wealth status of the population and the pattern of consumption of adequately iodized salt.

Regarding educational level, heads of households with higher education have a greater proportion of iodized salt consumption compared to those with lower levels of education. This aligns with studies conducted in Ethiopia, where individuals with formal education are twice as likely to consume iodized salt [22]. Additionally, it was found that people with higher and secondary education were 46% and 28% less likely, respectively, to consume salt without adequate iodine content [16]. In the same country, it was also found that women with formal education were 1.99 times more likely to consume adequately iodized salt [24]. When considering the ‘no education, preschool’ category, in contrast with studies conducted in Sub-Saharan Africa, it is evident that individuals without formal education or those who only attended preschool tend to have lower consumption of adequately iodized salt [39]. This may be due to the limited access to health information available through formal educational systems, making them more vulnerable to iodine deficiency. The lack of formal education restricts their knowledge about the benefits of iodized salt, highlighting the need for targeted public education interventions and access to mass media strategies that promote its consumption. To assess the population’s level of knowledge regarding iodized salt, a study was conducted in Ethiopia [22]. The people interviewed responded that they did not purchase iodized salt because they lacked adequate information and were unaware of its benefits. This lack of knowledge suggests that heads of households may not be aware of the benefits of consuming iodized salt and, therefore, may not prioritize its consumption. These results suggest that a higher level of education could improve knowledge about the importance and consumption of micronutrients. It is possible that educational level is associated with greater access to resources, which, in turn, could enhance both access to and the proper use of iodized salt in the population.

Regarding the type of residence, households in rural areas had a lower proportion of iodized salt consumption, which is consistent with previous studies [22,23,36]. This trend could be attributed to the fact that individuals with lower educational levels and limited economic resources tend to reside in rural areas, potentially placing them in a more vulnerable position concerning the consumption of adequately iodized salt [17,38]. It is important to highlight that the area of residence significantly influences the intake of essential micronutrients, such as vitamin A and iron, the deficiency of which severely compromises health and development at a global level [40]. Food insecurity disproportionately affects people living in rural areas [41], and the WHO indicates that rural populations have a higher prevalence of anemia [42]. For example, a study in the Kalalé district of Benin revealed that iron and vitamin A deficiencies are highly prevalent in this rural population, with 18.3% of women and 23.6% of children suffering from iron deficiencies, and 17.7% of women and 33.6% of children experiencing vitamin A deficiencies [43]. Similarly, a study conducted in 10 rural schools in Malaysia using children aged 7 to 11 found that 20.6% had a vitamin A deficiency, while the prevalence of anemia was 14.9%, being more common in children under 10 years old [44]. In contrast, in countries such as Uganda, no disparity was observed between urban and rural areas. This outcome is supported by previous studies in which the high proportion of adequately iodized salt consumption is attributed not only to universal salt iodization, but also significantly to the level of industrial consolidation and mechanization of the salt supply [20,23,36]. In this context, it would be particularly relevant for governments such as that of Uganda to share their successful practices with other nations, with the aim of illustrating the achievements in implementing salt iodization within their territory.

After analyzing these results and observing that the consumption of adequately iodized salt is directly related to wealth quintile, the education level of the household head, and area of residence, it could be assumed that countries with higher levels of poverty would be the most affected. However, there are successful cases of the implementation of universal salt iodization in countries such as Peru, which demonstrates that it is possible to overcome these challenges even in low-income contexts. Peru, having implemented universal salt iodization in 1986 [18], offers important lessons for other countries. Initially, various widely consumed foods, such as oil, were evaluated for fortification before salt was decided upon as the optimal vehicle for iodine supplementation. This effort included the creation of a national salt fortification policy, training for personnel responsible for salt production and distribution, and the establishment of a monitoring and quality control body, the ‘Centro Nacional de Alimentación, Nutrición y Vida Saludable’ (CENAN) [45]. A similar example is China, where, with strong political commitment and mandatory national legislation, universal salt iodization was initiated in 1960 [10]. China has successfully ensured access to iodized salt in 95% of its territory, with free distribution in hard-to-reach provinces, all supported by a rigorous national control and monitoring system. It is crucial for governments to understand the importance of iodine as an essential micronutrient for public health, which will enable long-term sustained commitment. This understanding must be translated into educational policies directed at the population, emphasizing the relevance of iodine consumption through salt. In this way, both household decision-makers and mothers can actively contribute to improving child development and choose better alternatives when purchasing. A relevant example is the rural areas of Wolaita, where the consumption of iodized salt is low. In interviews conducted there, 31.4% of respondents reported not consuming iodized salt due to a lack of knowledge about its benefits [22]. The successful experiences of Peru and China demonstrate that a strong political commitment, combined with comprehensive public education and robust monitoring systems, can result in effective and sustained iodized salt coverage. These examples highlight the need for governments to not only legislate universal salt iodization, but also ensure continuous public engagement through education, thereby fostering a culture of informed consumption.

Despite the significant global progress of the universal salt iodization program, Latin America and the Caribbean have shown uneven advancement. According to UNICEF, the lack of updated data limits the ability to generate accurate estimates of iodized salt coverage in regions such as Central Asia, the Middle East, North Africa, Latin America, and the Caribbean [13]. The lack of updated data, particularly in rural areas, limits the ability to assess the effectiveness of interventions. For example, natural disasters, as seen in Haiti, further complicate implementation and monitoring efforts, underscoring the vulnerability of health systems in these regions. To address these gaps, it is imperative to strengthen data collection mechanisms and establish more resilient monitoring frameworks [46].

It is essential to improve these mechanisms to ensure that iodization programs reach the most vulnerable populations equitably and sustainably. Furthermore, it is crucial to involve the food industry in this process by promoting the use of iodized salt in processed products that align with the cultural frameworks and dietary preferences of the population. However, for these strategies to be effective, political leaders must feel committed to the cause. The cost-effectiveness of salt iodization, with an estimated annual cost of USD 0.02–0.05 per child, is well established. This intervention not only prevents iodine deficiency, but also has long-term benefits for cognitive development, particularly in iodine-deficient populations [47]. The global impact is significant, with the cost per disability-adjusted life year gained estimated at USD 34–36. This underscores the critical importance of sustained political commitment to maintaining and expanding salt iodization programs, particularly in regions where coverage remains suboptimal [48].

Additionally, public education programs are crucial in raising awareness about the benefits of iodized salt consumption, especially in rural areas where misconceptions and lack of knowledge persist. Such initiatives should be integrated into broader public health campaigns, targeting both household decision-makers and key community leaders. Educational strategies that have proven successful in countries like Peru, where long-standing political commitment has facilitated sustained improvement in salt iodization coverage, could serve as models for other regions facing similar challenges.

This study has several limitations that should be considered. First, the cross-sectional nature of the study prevents the establishment of causal relationships. Additionally, the use of DHS data collected in different years introduces temporal variability. A methodological limitation is the use of rapid test kits for measuring iodine in salt, as these provide a qualitative assessment that may not be sufficiently precise enough to verify whether the iodine content meets established standards. It is also important to note that some study variables are based on self-reports from participants, which introduces the risk of information bias. Furthermore, the data may be affected by recall bias during interviews, which could influence the accuracy of the responses obtained. Finally, the significant heterogeneity observed in the meta-analysis (*I*^2^ = 100%) reflects considerable variability among the populations and contexts studied, which could limit the generalization of the findings to other settings. Nevertheless, the consistency in the format and methodology of the DHS surveys facilitates the comparability of the information collected across countries.

## 5. Conclusions

Although approximately 80% of households globally consume adequately iodized salt, the figures vary considerably across different regions, socioeconomic statuses, education levels, and places of residence. Regions such as Sub-Saharan Africa and Central Asia present a high proportion of households consuming adequately iodized salt, reflecting more successful efforts in implementing iodization policies. In contrast, Latin America and the Caribbean present significantly lower rates, highlighting the urgent need to strengthen interventions in these areas. Disparities become even more evident when analyzing socioeconomic factors and education levels. Households with higher wealth and education, especially those in urban areas, are more likely to consume adequately iodized salt. On the other hand, more vulnerable populations, such as households with lower incomes and lower educational levels, as well as those in rural areas, face limited access to this micronutrient. This demonstrates that current iodization policies are not equitably reaching all segments of society, perpetuating health inequalities within countries.

It is crucial for health policymakers to address these inequities by implementing targeted strategies that consider the socioeconomic, educational, and geographical particularities of each region. Iodine deficiency, especially in the early years of life, can have severe consequences for cognitive and physical development, perpetuating cycles of poverty and disease. Addressing these disparities is essential for ensuring equitable access to health and preventing long-term adverse effects on the population.

## Figures and Tables

**Table 1:** DHS surveys by country and year.

Country	World Bank Region	Survey Year	Total Households Included
Papua New Guinea	East Asia and Pacific	2017	4564
Cambodia	East Asia and Pacific	2014	15,622
Myanmar	East Asia and Pacific	2016	12,291
Timor-Leste	East Asia and Pacific	2016	11,359
Kyrgyz Republic	Europe and Central Asia	2012	7928
Tajikistan	Europe and Central Asia	2017	7806
Moldova	Europe and Central Asia	2005	10,769
Albania	Europe and Central Asia	2017	15,341
Armenia	Europe and Central Asia	2016	7838
Azerbaijan	Europe and Central Asia	2006	7118
Haiti	Latin America and Caribbean	2016	12,400
Guyana	Latin America and Caribbean	2009	5229
Peru	Latin America and Caribbean	2021	22,786
Egypt	Middle East and North Africa	2014	9099
Yemen	Middle East and North Africa	2013	16,387
Afghanistan	South Asia	2015	23,690
Nepal	South Asia	2016	10,929
Burundi	Sub-Saharan Africa	2016	14,389
Comoros	Sub-Saharan Africa	2012	3892
Kenya	Sub-Saharan Africa	2014	34,139
Malawi	Sub-Saharan Africa	2015	22,489
Mozambique	Sub-Saharan Africa	2011	13,139
Tanzania	Sub-Saharan Africa	2015	11,732
Zambia	Sub-Saharan Africa	2018	11,485
Zimbabwe	Sub-Saharan Africa	2015	8056
Uganda	Sub-Saharan Africa	2016	17,851
Angola	Sub-Saharan Africa	2015	14,269
Chad	Sub-Saharan Africa	2014	15,881
Congo	Sub-Saharan Africa	2011	10,453
Congo Democratic Republic	Sub-Saharan Africa	2013	16,076
Gabon	Sub-Saharan Africa	2012	8712
Sao Tome and Principe	Sub-Saharan Africa	2008	2560
Eswatini	Sub-Saharan Africa	2006	4509
Lesotho	Sub-Saharan Africa	2014	6583
Namibia	Sub-Saharan Africa	2013	9196
South Africa	Sub-Saharan Africa	2016	2443
Benin	Sub-Saharan Africa	2017	13,288
Burkina Faso	Sub-Saharan Africa	2010	13,713
Cote d’Ivoire	Sub-Saharan Africa	2012	8503
Gambia	Sub-Saharan Africa	2019	5630
Ghana	Sub-Saharan Africa	2014	10,237
Guinea	Sub-Saharan Africa	2018	7496
Liberia	Sub-Saharan Africa	2019	7956
Mali	Sub-Saharan Africa	2018	9106
Mauritania	Sub-Saharan Africa	2020	10,309
Niger	Sub-Saharan Africa	2012	9008
Nigeria	Sub-Saharan Africa	2018	37,780
Sierra Leone	Sub-Saharan Africa	2019	12,152
Togo	Sub-Saharan Africa	2013	8957

## Data Availability

Zenodo Repository: https://zenodo.org/records/13370386.

## Supplementary Materials

The following supporting information can be downloaded at: https://www.mdpi.com/article/10.3390/nu16213787/s1, Figure S1: Sensitivity Analysis.
