# Supplementary figures and images for: Household Consumption of Adequately Iodized Salt: A Multi-Country Analysis of Socioeconomic Disparities
Source: Nutrients. 2024 Nov 4;16(21):3787. doi: 10.3390/nu16213787 (PMC11547564; doi:10.3390/nu16213787)

Figure S1: Sensitivity Analysis

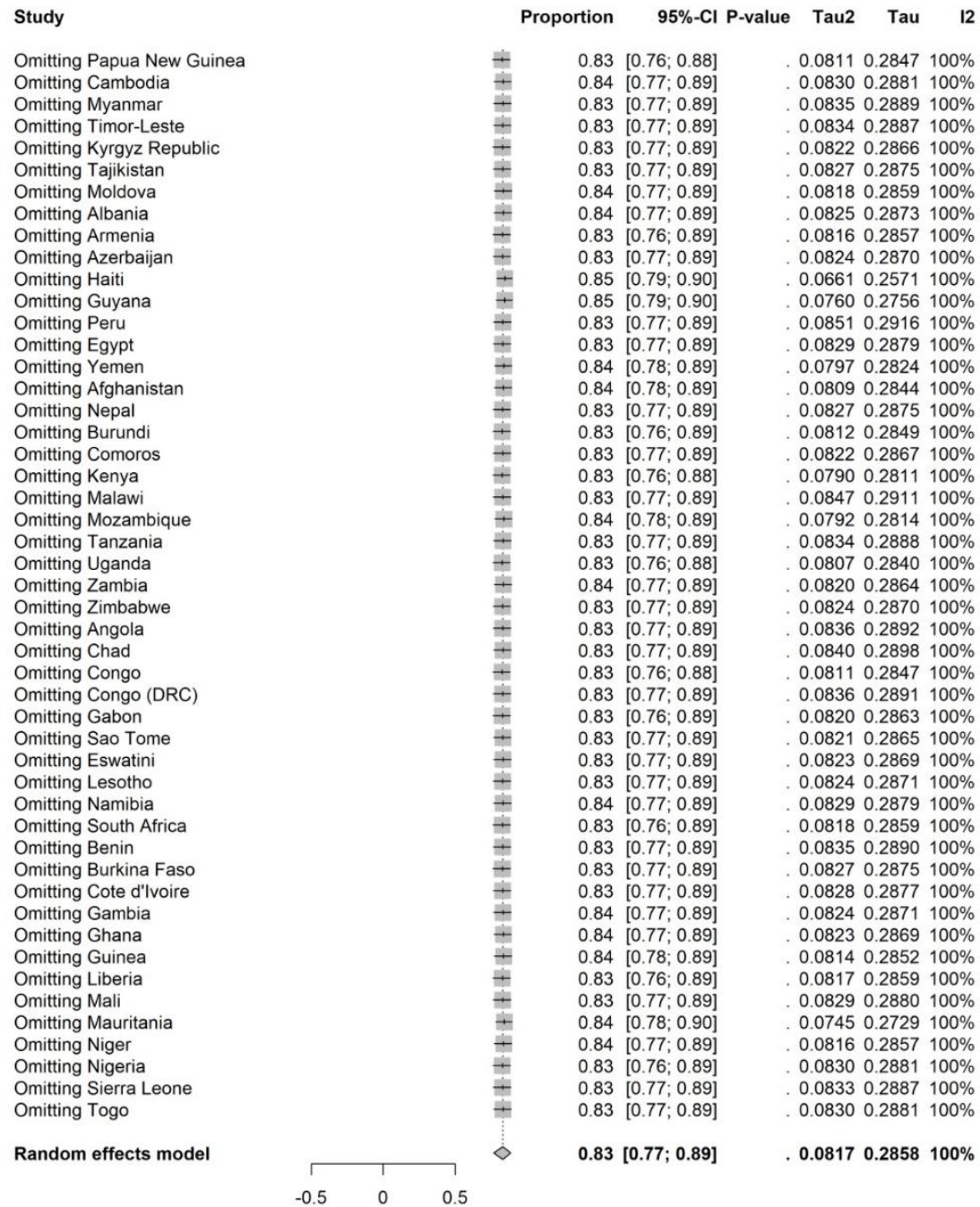

Supplement: Supplementary file 1 [file nutrients-16-03787-s001.zip › nutrients-3230557-supplementary.pdf]
